# Robust Assessment of Free-Living Physical Behaviors and Activity Intensity Using Dual-Wearable Multitask Learning: Development and Evaluation Study From the Multicenter WEALTH Project

**DOI:** 10.2196/94302

**Published:** 2026-08-14

**Authors:** Luis Sigcha, Annika Swenne, Grainne Hayes, Jitka Kuhnova, Richard Cimler, Steriani Elavsky, Tomas Vetrovsky, Léopold Fezeu Kamedjie, Jérôme Bouchan, Jean-Michel Oppert, Janas Harrington, Greet Cardon, Antje Hebestreit, Alan Donnelly, Pepijn Van de Ven, Christoph Buck, WEALTH consortium, Alan Donnelly

**Affiliations:** 1 Centro ALGORITMI/LASI, School of Engineering, University of Minho, Campus de Azurém, University of Minho, Guimaraes, 4800-058, Portugal, 34 640177812; 2 Data-Driven Computer Engineering (D2iCE) Research Centre, Department of Electronic and Computer Engineering, University of Limerick, Limerick, Munster, Ireland; 3 Health Research Institute, University of Limerick, Limerick, Ireland; 4 Leibniz Institute for Prevention Research and Epidemiology - BIPS, Bremen, Germany; 5 Faculty of Mathematics and Computer Science, University of Bremen, Bremen, Germany; 6 Department of Physical Education and Sport Sciences, University of Limerick, Limerick, Ireland; 7 Faculty of Science, University of Hradec Kralove, Hradec Kralove, Czech Republic; 8 Department of Human Movement Studies, University of Ostrava, Ostrava, Czech Republic; 9 Faculty of Physical Education and Sport, Charles University, Prague, Czech Republic; 10 INSERM U1153, INRAE U1125, CNAM, Centre de Recherche en Epidémiologie et Statistiques (CRESS) Équipe de Recherche en Épidémiologie Nutritionnelle (EREN), Université Sorbonne Paris Nord et Université Paris Cité, Bobigny, France; 11 Department of Nutrition, Pitie-Salpetriere hospital (AP-HP), Sorbonne Université, Paris, France; 12 HRB Centre for Health and Diet Research, School of Public Health, University College Cork, Cork, Ireland; 13 Department of Movement and Sports Sciences, Ghent University, Ghent, Belgium; 14 See Acknowledgments

**Keywords:** accelerometers, convolutional neural networks, deep learning, human activity recognition, physical activity, sedentary behavior

## Abstract

**Background:**

Accurate assessment of physical behaviors (PBs) and activity intensity is essential for public health research and digital health monitoring. Wearable accelerometers combined with machine learning (ML) or deep learning (DL) enable objective behavior assessment, but most existing models are trained on laboratory data, limiting generalizability to free-living conditions.

**Objective:**

This study aimed to develop and evaluate multitask ML and DL models for PB classification across 7 categories (sitting, standing, walking, running, sports, cycling, and lying) and activity intensity categories (AIC) across 3 levels (sedentary, light, and moderate-to-vigorous physical activity) using thigh-worn (activPAL) and waist-worn (ActiGraph) wearable accelerometers. A second objective was to compare model-derived estimates of daily time spent in PB and AIC across single- and dual-sensor (activPAL + ActiGraph) configurations, and to evaluate agreement between the best-performing model and corresponding estimates obtained from the proprietary activPAL classification of real-world everyday activities (CREA) algorithm using free-living data collected over a 9-day monitoring period.

**Methods:**

Data were obtained from 590 adults in the multicenter WEALTH study (627 recruited) and included up to 9 days of concurrent activPAL and ActiGraph free-living recordings. Sparse accelerometer-labeled data were obtained using ecological momentary assessment and refined by retaining instances with ≥75% agreement with the CREA algorithm. Resulting labeled data of 583 participants were used to develop ML models for single-sensor (activPAL or ActiGraph) and combined (dual-sensor) configurations. A random forest (RF) model using engineered features and a multihead convolutional neural network (MH-CNN) were trained within a multitask learning framework to jointly predict PB (task 1) and AIC (task 2) using a subject-independent hold-out split. The test subset (n=87) was used to estimate daily time spent in PB and AIC over 9 days, which were compared with CREA-derived estimates using Pearson coefficients and intraclass correlation coefficients (ICCs).

**Results:**

The dual-sensor configuration consistently outperformed single-sensor models. For PB classification, the MH-CNN achieved the highest performance (*F*_1_-score=0.750). For AIC, the RF model performed best (*F*_1_-score=0.741). Dual-sensor free-living estimates showed epidemiologically plausible distributions across the 24-hour period, including sitting 37% (538/1440 min), lying 34% (496/1440 min), walking 9% (131/1440 min), and moderate-to-vigorous physical activity (MVPA) 2% (31/1440 min). Agreement with CREA was strongest for standing, walking, and cycling (*r*≥0.86; ICC ≥0.72), while lying showed modest reliability (ICC=0.48). For AIC, agreement was highest for light physical activity (LPA) and MVPA (ICC 0.72‐0.75).

**Conclusions:**

Multitask models combining thigh- and waist-worn accelerometers provide consistent estimates of PB and AIC under free-living conditions. The dual-sensor approach yielded more stable and epidemiologically coherent estimates than single-sensor methods, supporting its potential for large-scale population monitoring and mobile health apps.

## Introduction

Robust assessment of movement and posture in free-living conditions is fundamental to population surveillance, behavioral interventions, and remote health monitoring. Recent advances in mobile health (mHealth) apps and wearable technologies have enabled more objective and scalable measurement beyond laboratory or clinical environments. However, variability in measurement approaches continues to limit comparability across studies and applications [1-3]. Consequently, there is a clear need for reliable, standardized methods capable of capturing physical behavior (PB) and activity intensity in real-world settings.

In this context, PB refers to posture- and movement-based activity types that describe how time is accumulated across the day, including sedentary postures (eg, sitting or lying), upright postures (eg, standing), and movement behaviors (eg, walking, running, or cycling) [4]. Activity intensity complements PB by characterizing the intensity associated with these behaviors. In population-based research, activity intensity is commonly operationalized using metabolic equivalents of task (METs), which can be used to estimate energy expenditure [5,6]. More commonly, METs are categorized into activity intensity categories (AIC), including sedentary behavior, light physical activity (LPA), and moderate-to-vigorous physical activity (MVPA), using established cut-points from the Compendium of Physical Activities [7] (eg, sedentary behavior 1.0‐1.5 METs; LPA 1.6‐2.9 METs; MVPA ≥3.0 METs), supported by consensus definitions in the sedentary behavior literature [8].

Wearable accelerometers have become central to mHealth research because they enable continuous monitoring of physical activity (PA) and sedentary behavior under free-living conditions [9]. Thigh-worn devices such as the activPAL have demonstrated strong validity for posture classification, including sitting, standing, stepping, and cycling, owing to their anatomical placement and compatibility with 24-hour wear protocols [4,10]. In contrast, waist-worn accelerometers such as the ActiGraph monitor are widely used in population-based studies and provide reliable estimates of ambulatory activity and overall activity volume [11]. ActiGraph devices are among the most widely adopted wearable accelerometers for device-based assessment of PB in free-living settings. However, consistent with previous validation studies, their ability to distinguish postures, particularly standing from sitting, is more limited, reflecting the reduced sensitivity of waist-mounted accelerometers to subtle changes in trunk orientation [12-14]. These limitations can result in systematic discrepancies in daily PB and AIC estimates, thereby complicating interpretation and comparability in mHealth apps.

Recent advances in machine learning (ML) and deep learning (DL) methods have substantially improved the accuracy and scalability of human activity recognition using wearables. These methods excel at learning complex temporal and biomechanical patterns that are difficult to capture with traditional rule-based algorithms [15,16]. Among these advances, multitask learning has emerged as a particularly promising framework [17]. By allowing a single model to learn shared representations across related outcomes, such as PB and AIC classification, multitask learning leverages complementary information embedded in accelerometer signals. This joint learning strategy can improve model stability, reduce overfitting, and enhance robustness in free-living environments, where movement patterns are highly variable, context-dependent, and often noisy [18]. Additionally, multihead convolutional neural networks (MH-CNNs) are increasingly used for accelerometer-based movement analysis, extending standard architectures through parallel processing heads that learn complementary representations and support the simultaneous modeling of heterogeneous signal characteristics, such as different temporal scales and movement patterns [19,20].

The limited availability of reliable, ecologically valid, and accurately labeled datasets remains a critical barrier to the development of robust ML models for population-level studies. Many existing approaches rely on data collected using laboratory-based protocols or short-duration recordings, which fail to capture the diversity and complexity of real-world behavior [21,22]. In this context, ecological momentary assessment (EMA) offers a scalable alternative by collecting self-reported behavioral information in real time, reducing recall bias and improving temporal alignment with sensor data [23]. When combined with wearable sensing, EMA enables the generation of labeled free-living datasets that more accurately reflect naturalistic behavior patterns, supporting the development of models with improved ecological validity [24].

Despite these advances, relatively few studies have combined long-term free-living monitoring, multimodal wearable sensing, and subject-independent validation at scale. Large, labeled datasets such as CAPTURE-24 provide high-quality annotations but are limited in duration [25], while other resources offer contextual information but provide limited temporal resolution or constrained sensor configurations [26]. Moreover, the added value of combining posture-sensitive and movement-sensitive sensors for improving the stability and interpretability of daily PB and AIC estimates remains insufficiently quantified [27].

To address this gap, the wearable sensor assessment of physical and eating behaviors (WEALTH) project integrated standardized laboratory protocols with a 9-day free-living monitoring period across European populations, complemented by event-based (sensor-triggered) EMA delivered via the HealthReact mobile app [28]. These data enabled the generation of sparse (EMA) labeled accelerometer data, further refined using agreement with activPAL’s proprietary classification of real-world everyday activities (CREA) algorithm, resulting in a large free-living dataset with quality-controlled labels [23].

Against this background, the main objective of this study was therefore to develop and evaluate multitask ML and DL models for simultaneous PB classification (task 1) and AIC classification (task 2) using thigh- and waist-worn accelerometry, both individually and in a combined (dual-sensor) configuration, under free-living conditions. In support of this objective, multimodal sensing and EMA-informed labels were integrated to facilitate robust behavioral monitoring in ecologically diverse settings. Subsequently, the best-performing models were used to estimate continuous PB and AIC over 9 days. These outputs were aggregated to derive daily time spent in each activity and AIC, enabling the comparison of behavioral patterns across sensor configurations (activPAL, actiGraph, and dual-sensor) and against CREA-derived daily estimates to assess agreement, stability, and ecological coherence. By focusing on ecological validity, scalable data collection, subject-independent validation, and real-world deployment considerations, this work aims to advance methodological foundations for population monitoring in free-living settings, while providing detailed estimates of daily PB and AIC profiles.

## Methods

### Study Design

This study used data from the WEALTH project, a large, multicenter cohort conducted in Czechia, France, Germany, and Ireland. The study supported the development of standardized methods for assessing daily PA, sedentary behavior, and eating behaviors by integrating structured laboratory assessments with free-living monitoring to ensure ecological validity.

Participants first attended a laboratory session involving a 75-minute semistructured activity protocol, during which well-defined movement patterns were recorded under controlled conditions using 4 different sensors including activPAL 3 micro (PAL Technologies), ActiGraph GT3X (ActiGraph LLC), Skagen Falster Gen 5 smartwatch (Skagen, Fossil Group Inc), and a Fitbit Charge 5 (Fitbit Inc., Google LLC). This was followed by a 9-day free-living phase, during which the 4 different sensor devices collected accelerometer data in combination with event-based EMA to capture contextual information about participants’ behaviors in real-world settings [29].

### Participants

A cohort of 627 adults was recruited across 4 European study sites using a convenience sampling strategy through university, community, and institutional advertisement channels, resulting in a selective but demographically diverse sample. Sample size was determined according to the WEALTH study protocol [29]. The target sample was selected to support machine-learning model development and to accommodate expected data loss arising from device noncompliance and data quality exclusions. Participants were required to have no mobility impairments and to be able to complete both the laboratory and free-living protocols. From the initial cohort, 32 participants did not provide concurrent activPAL and ActiGraph recordings, and an additional 5 participants were excluded due to significant temporal drift that precluded reliable sensor synchronization. The final sample consisted of 590 participants with valid and concurrent activPAL and ActiGraph data. Participant characteristics are summarized in Table 1 and reflect variability in age, sex, country, and BMI, including representation of individuals classified as obese (BMI ≥30 kg/m²).

**Table 1.** Demographic information of the participants included in this study (N=590).

Characteristics	Values
Age (years), mean (SD)	38.1 (14.3)
Height (cm), mean (SD)	171.2 (9.9)
Weight (kg), mean (SD)	72.0 (14.3)
BMI (kg/m²), mean (SD)	24.5 (4)
Obesity (BMI ≥30 kg/m²), n (%)	54 (9.2)
Country, n (%)	
Czechia	138 (23)
France	139 (24)
Germany	157 (27)
Ireland	156 (26)
Sex, n (%)	
Male	258 (44)
Female	332 (56)

### Wearable Sensor Configuration and Data Acquisition

A combination of research-grade and consumer-grade wearable devices was used to capture movement patterns, posture, and activity-related events throughout both the laboratory session and the 9-day free-living period. As part of this multimodal setup, an activPAL 3 micro was placed on the midline of the anterior aspect of the right thigh using a hypoallergenic adhesive tegaderm dressing. The device recorded continuous triaxial acceleration at 20 Hz, provided estimates of activity intensity expressed as METs, and behavioral outputs through its proprietary CREA algorithm, classifying sedentary, standing, stepping, cycling, and nonwear, which offered a reference for refining EMA-derived annotations.

To complement the thigh-worn sensor, participants also wore an ActiGraph affixed to the right waist. This device provided higher-frequency (100 Hz) movement data, which contributed to the development of dual-sensor models by enabling an additional perspective on upper-limb and whole-body movement patterns.

Finally, participants wore a Fitbit Charge 5 on the nondominant wrist to trigger event-based EMA surveys. Although Fitbit data were not used as model input, step count and heart rate data were used to detect predefined behavioral conditions and automatically initiate EMA prompts. An overview of the body-worn wearables, data sources, and processing workflow used in this study is provided in Multimedia Appendix 1.

### EMA

EMA assessments were implemented using the HealthReact mobile app [28], which delivered three types of surveys throughout the 9-day free-living period: (1) event-based (sensor-triggered) surveys, (2) time-based surveys, and (3) participant-initiated surveys. Only data from event-based surveys were used, as they were automatically triggered in response to specific sensor-detected activities relevant to this study.

Event-based survey prompts were generated automatically based on Fitbit-recorded data, triggering an EMA survey after ≥20 minutes of zero steps for sedentary behavior, ≥5 minutes of walking at 60‐139 steps/minute, or ≥5 minutes of running at ≥140 steps/minute. Participants reported the activity and posture being performed, while timestamps were automatically logged by the HealthReact app. Prompt allocation rules were pilot-tested following Janek et al [23], with up to 4 daily prompts for sedentary behavior and up to 3 each for walking and running.

### Data Synchronization and Preprocessing

The raw accelerometer data were processed using ActiLife (v6.8) for the ActiGraph devices and PAL Analysis (v9.1) for the activPAL devices. For activPAL, the activity annotations and MET-based activity intensity estimates (expressed in MET-seconds) were extracted using PAL Analysis software and the proprietary CREA algorithm. An epoch length of 60 seconds was applied to ensure consistency with prior studies and established protocols for MET-based activity intensity assessment [30,31].

To harmonize the recordings across devices, all acceleration signals were expressed in gravitational units (g). ActiGraph data (100 Hz) were resampled to 20 Hz to match activPAL’s native frequency. Initial synchronization was performed using device timestamps and the recorded start of the laboratory session. A fine-grained alignment was subsequently performed and verified by visual inspection using MATLAB (R2020a). Temporal offsets attributable to internal clock drifts were compensated by inserting a small, fixed number of samples (80 samples per day) into the ActiGraph data at a daily interval. This correction accounted for systematic clock drift between activPAL and ActiGraph devices during prolonged free-living recordings, which can produce cumulative timing misalignment between posture and activity signals despite simultaneous initialization [32].

Following device-level synchronization, the CREA-derived MET estimates were temporally aligned with accelerometer time-series. Activity intensity estimates were converted from MET-seconds to MET values and subsequently mapped to categorical intensity. In this study, AIC were operationalized using MET-derived categories rather than direct kcal-based energy expenditure estimates to enable standardized intensity classification for population-level free-living analyses. This enabled classification into sedentary behavior (1.0‐1.5 MET), LPA (1.6‐2.9 MET), and MVPA (≥ 3 MET) categories using standard MET-based cut-points [7,8].

### EMA-Based Sparse Labeling and Quality-Controlled Segment Selection

Sparse free-living labeled data were generated following the EMA-based labeling framework proposed in Sigcha et al [24]. During the 9-day data collection period, 26,166 EMA activity surveys were recorded. After removing duplicates and surveys lacking valid posture or activity information, 16,907 surveys were retained for wearable data annotation. EMA triggers were used to segment the continuous accelerometer data streams, and each resulting data segment was labeled using the participant’s response to the associated EMA prompt.

The resulting segments were annotated according to predefined PB categories established within the WEALTH study: sitting, standing, walking, running, sports, cycling, and lying, resulting in temporally sparse but highly specific annotations. This EMA-based approach prioritizes ecological validity and label specificity over label density, thereby providing high-quality annotations under free-living conditions.

To strengthen label reliability, only segments showing at least 75% sample-wise agreement with CREA were retained for model training and evaluation. In line with the findings reported in [24], this threshold was selected as an appropriate trade-off between data quality and quantity, reducing less confident segments while preserving sufficient free-living coverage across activity types, ensuring that the final labeled dataset was both ecologically valid and of high quality.

Following EMA-based annotation and quality control using a ≥75% agreement threshold with the CREA algorithm, 2108 hours of labeled free-living data from 583 participants were retained for model development and validation. The reduction in the number of participants from 590 to 583 was primarily due to EMA surveys that could not be synchronized with the corresponding accelerometer recordings because of time-stamp mismatches, which placed the EMA entries outside the valid recording range and prevented reliable segment construction. Detailed distributions of the EMA-labeled data before and after quality control are provided in Multimedia Appendix 2.

### Generation of Unlabeled Dual-Sensor Datasets for Model-Derived PB and AIC Estimates

In addition to EMA-labeled data, the 9-day free-living (unlabeled) recordings from activPAL and ActiGraph devices were synchronized to enable the implementation and evaluation of a dual-sensor approach for generating model-derived daily PB and AIC estimates, as well as their corresponding 9-day averages. To ensure strict temporal correspondence between the 2 devices, each pair of recordings was then trimmed to match the sensor file with the lowest sample count and visually inspected using MATLAB.

Following this synchronization procedure, complete unlabeled free-living data were obtained from 590 participants (Table 1), representing the full 9-day monitoring period and providing the basis for subsequent analyses of daily PB and AIC profiles.

### Data Segmentation and Feature Extraction

To prepare the data for model development, raw time-series signals were segmented using nonoverlapping windows. Multiple window lengths (5, 10, and 15 s) were evaluated to determine their influence on classification performance, reflecting common practices in human activity recognition [24,25]. For each window, a PB label (task 1) and AIC label (task 2) were assigned using 2 different approaches. The PB label was directly taken from the EMA-reported activity for the corresponding prompt, reflecting the participant’s self-reported behavior within that data segment. In contrast, because MET-based estimates were available at a 60-second epoch resolution, a majority voting technique [33] was applied to assign a single AIC label to each window. Specifically, each window was labeled as Sedentary, LPA, or MVPA based on the AIC most frequently observed in the specific window.

Three feature-extraction strategies were implemented to support classical ML pipelines. First, a set of 40 features was computed following the CAPTURE-24 specification [25], capturing statistical, spectral, and temporal characteristics. Second, an adapted version of the HARTH feature set [34] was generated for single-sensor use, extending its applicability beyond the original dual-sensor design. Third, the original HARTH feature set [34] was extracted for dual-sensor configurations to leverage complementary information from thigh- and wrist-based measurements. A detailed description of the set of features extracted in this study is provided in the Multimedia Appendix 3.

In parallel, raw accelerometer data were used to train a multihead convolutional neural network (MH-CNN), implemented in both single- and dual-sensor variants. This DL-based approach enabled the extraction of hierarchical temporal patterns directly from the raw signals, providing an alternative to feature-engineered models.

### ML and DL Models

#### Overview

To evaluate both ML and DL methods, 2 multitask models were developed: a feature-based random forest (RF) model and the MH-CNN. These models were designed to jointly predict PB (task 1) and AIC (task 2) using triaxial accelerometer data.

#### RF

The RF model was selected for its robust performance in PA recognition tasks and its relatively small number of hyperparameters, which facilitates efficient optimization and reduces the risk of overfitting. Separate RF models were trained for each feature set described in the previous section, with each feature set providing a structured representation of the underlying accelerometer signals.

#### MH-CNN

**Figure 1.**
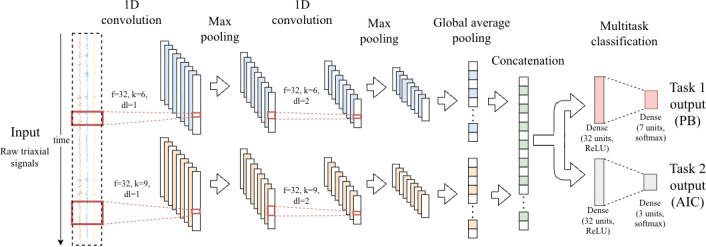
Architecture of the multihead convolutional neural network adapted for multitask classification. AIC: activity intensity categories; dl: dilatation rate; f: number of filters; k: kernel size; PB: physical behavior; ReLU: rectified linear unit.

An adapted MH-CNN [19,20] was implemented to assess the performance of a DL-based approach trained directly on raw accelerometer data. The architecture was selected to balance classification performance, computational efficiency, and scalability for large-scale applications, while reducing the computational burden associated with more demanding sequential architectures. The network architecture comprised 2 parallel convolutional branches with distinct kernel sizes (eg, 9 for a large kernel and 6 for a medium-sized kernel), designed to capture both fine- and coarse-grained temporal dynamics. Each branch included 2 convolutional layers (32 filters each) with max pooling, followed by global average pooling. The extracted features were then passed to separate fully connected (dense) layer branches to support multitask classification. Figure 1 shows the architecture of the MH-CNN with 2 heads, with the output adapted for multitask learning.

Hyperparameter optimization was performed for both models. For the RF classifier, optimization was carried out using grid search cross-validation, which enabled an exhaustive evaluation of key parameters while keeping computational demands manageable. For the MH-CNN, the Hyperband algorithm [35] was used to efficiently explore a broad space of architectural and training configurations. Details on the parameters and ranges used for the optimization of these models are included in the Multimedia Appendix 4.

To accelerate the optimization process, both procedures were conducted using laboratory data from a reduced subset of 100 participants, as prior work suggested limited performance gains beyond this sample size [25]. Laboratory data were used for model optimization, providing a controlled setting that allowed consistent tuning while avoiding the added variability inherent to free-living data during this stage of model development.

After the optimization, the RF model used for training had 150 decision trees, a maximum tree depth of 10, and a maximum of 16 features considered in each split. The resulting parameters for the MH-CNN are shown in Figure 1 and detailed in the Multimedia Appendix 5.

### Model Training and Evaluation

To ensure a robust assessment of model performance, a subject-independent hold-out strategy was implemented using the EMA-labeled dataset. Of the original cohort (N=590) participants, data from 583 individuals were available for model training after excluding cases where EMA responses could not be synchronized with accelerometer data. The resulting participants were allocated to training (407/583, 70%), validation (87/583, 15%), and test (87/583, 15%) subsets, with each individual assigned to only 1 data subset to prevent information leakage across subsets. Stratification by age, gender, and country preserved demographic balance across subsets to support a reliable model evaluation. The number of participants and the characteristics of the data subsets used to train and evaluate the ML models are detailed in the Multimedia Appendix 6.

**Figure 2.**
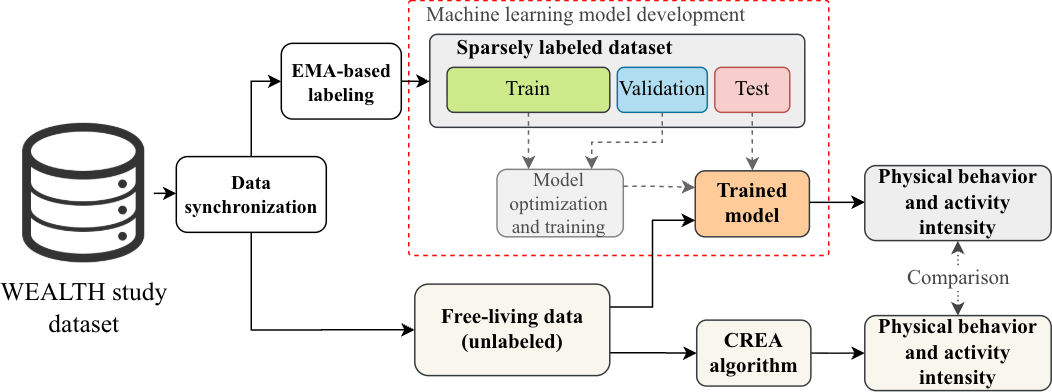
Overall methodology for machine learning model development and agreement analysis. CREA: classification of real-world everyday activities; EMA: ecological momentary assessment.

Model performance for both PB and AIC classification was quantified using classification metrics, including precision, recall, and *F*_1_-score [36]. These complementary indicators allowed for a comprehensive evaluation across the imbalanced activity classes. Figure 2 presents the overall methodology for ML model development and agreement analysis.

ML and DL models were trained on a workstation equipped with an Intel Xeon 2.30 GHz processor, 25 GB RAM, and a 12 GB NVIDIA Tesla K80 GPU. ML models were implemented in Python (v3.12) using Pandas (v2.2), NumPy (2.0.2), and Scikit-learn (v1.6). To address class imbalance, the ML training subset was undersampled so that all activity classes matched the size of the smallest class. Undersampling was chosen because the dataset is highly skewed toward a single category; RF models can be biased by class imbalance; it also reduces training time, mitigates the risk of overfitting, and avoids potential artifacts introduced by synthetic data generation techniques such as the synthetic minority oversampling technique (SMOTE).

DL models were developed using Keras (3.10) and TensorFlow (2.19). Unlike the ML pipeline, undersampling for the DL model was applied selectively by reducing only the most frequent classes (sitting to 30,000 samples; lying to 25,000; walking to 20,000) to preserve the natural distribution of daily activities while controlling class dominance. As with ML, undersampling was performed exclusively on the training data subset.

The MH-CNN architecture was trained using a multitask learning framework with task-specific weighting prioritizing PB over AIC classification (ratio 10:1), together with class-weighted losses and early stopping to reduce overfitting. Optimization was performed using the adaptive moment estimation (ADAM) optimizer [37]. Additional details regarding training configuration and procedures are provided in Multimedia Appendix 5.

### ML Experiments

Different experiments were conducted to evaluate the effects of window size, sensor type, and sensor configuration on PB and AIC classification.

The effect of window size was evaluated by segmenting synchronized accelerometer data into nonoverlapping windows of 5, 10, and 15 seconds. These analyses were performed using the dual-sensor configuration to identify an appropriate temporal resolution for subsequent experiments.

Single-sensor experiments were then carried out separately for activPAL and ActiGraph. For each device, RF models trained with hand-crafted features (CAPTURE-24 and an adapted single-sensor HARTH feature set) were compared with MH-CNN trained on raw accelerometer signals. This design allowed for a direct comparison between feature-engineered ML approaches and end-to-end DL models.

Finally, dual-sensor experiments were performed by combining activPAL and ActiGraph data. In this setting, RF models were trained using the original (dual-sensor) HARTH feature set and compared with the MH-CNN trained on synchronized raw data from both sensors.

In all experiments, PB and AIC classification were treated as separate outcomes but evaluated within the same experimental framework. Results of these experiments are reported as mean and SD across 5 repeated runs to identify the model stability during training. In addition, bootstrap resampling (n=100) was used to estimate 95% CIs for the *F*_1_-scores across the evaluated window lengths following the methodology proposed by Efron [38].

### Evaluation of Model-Derived Daily PB and AIC Profiles

#### Overview

Following ML development, the best-performing models were applied to unlabeled (free-living) data to estimate daily time spent in distinct PBs and AIC. The evaluation comprised two complementary analyses: (1) the derivation of model-based daily PB and AIC profiles across different sensor configurations, and (2) an agreement analysis between the best-performing model and the activPAL CREA algorithm. All analyses were conducted using free-living data from 87 participants not included in model training or optimization, ensuring an unbiased assessment of generalizability.

#### Model-Derived Daily PB and AIC Profiles Across Sensor Configurations

In the first analysis, model-derived daily PB and AIC profiles were generated separately for three sensor configurations: activPAL, ActiGraph, and the combined dual-sensor setup. Valid monitoring days were defined as those with ≥8 hours of wear time [39-41]. Participant-level summaries were calculated using a weekday-weekend weighting scheme (5/7 weekdays, 2/7 weekend days) [42], and participants were included if they met the 3-day validity criterion (at least 2 valid weekdays and 1 valid weekend day).

For both PB and AIC, weighted participant-level estimates were subsequently summarized across participants to characterize population-level daily profiles. Variability in estimates across participants was quantified using 95% CIs, calculated via a normal approximation based on the sample mean, sample SD, and the number of participants contributing valid data to each estimate.

#### Comparison of Dual-Sensor Estimates With CREA

In the second analysis, the agreement between the best-performing model (dual-sensor configuration) and the activPAL CREA-derived estimates was evaluated. To ensure compatibility between the 2 systems, CREA activity labels “primary lying” and “secondary lying” were merged into a single lying category, enabling direct comparison of PB categories common to both approaches (eg, standing, walking, cycling, and lying). No additional harmonization was required for AIC, as both methods classify intensity using the same 3 categories (sedentary, LPA, and MVPA).

Unlike the sensor-configuration analysis described above, this comparison was performed at the daily level without applying weekday-weekend weighting to enable a direct, day-by-day comparison with CREA outputs. This approach preserved the original temporal structure of the CREA estimates and avoided introducing additional aggregation effects that could mask day-level agreement between methods.

Agreement was evaluated using correlation plots and Bland-Altman analyses to visually assess the relationship and systematic bias between methods. In addition, Pearson correlation coefficients (*r*; 2-sided α=.05) were used to quantify the strength of association, and intraclass correlation coefficients (ICC [1,2]) were computed and reported with 95% CIs to evaluate absolute agreement between methods at the daily level.

### Ethical Considerations

Prior to the study’s commencement, ethics committee approval was obtained from all participating institutions: The Education and Health Sciences Faculty Research Ethics Committee, University of Limerick (22_09_10_EHS); the Ethics Committee of the University of Bremen (2022‐25); the Comité de Protection des Personnes CPP Île-de-France VI, Paris (2022-A02208-35); and the Committee for Research Ethics at the University of Hradec Králové, Czech Republic (11/2022). All participants provided written informed consent before data collection and were informed of their right to withdraw from the study at any time without providing justification. To acknowledge participants’ time and contribution, monetary compensation was provided: €40 in Ireland and €20 in the other countries. All collected data were deidentified for analysis and stored securely to maintain participant confidentiality.

## Results

### Model Development and Classification Performance

#### PB and AIC Classification Performance Across Window Lengths

The impact of window size on model performance was evaluated using the dual-sensor configuration by combining the HARTH (dual-sensor) feature set with an RF algorithm. Table 2 shows the results of these experiments.

**Table 2.** Physical behavior and activity intensity categories classification performance across window lengths using the dual-sensor random forest model.

Window size (seconds)	Precision	Recall	*F*_1_-score
PB^a^, mean (SD)			
5	0.675 (0.005)	0.821 (0.004)	0.729 (0.004)
10	0.671 (0.002)	0.825 (0.002)	0.729 (0.002)
15	0.667 (0.007)	0.828 (0.005)	0.727 (0.006)
AIC^b^, mean (SD)			
5	0.736 (0.0004)	0.737 (0.001)	0.728 (0.001)
10	0.743 (0.002)	0.749 (0.001)	0.741 (0.002)
15	0.744 (0.001)	0.754 (0.002)	0.742 (0.001)

a PB: physical behavior.

b AIC: activity intensity categories.

PB classification performance was comparable across window lengths, with similar *F*_1_-scores for 5- and 10-second windows (both 0.729) and a marginal reduction for 15-second windows (0.727). AIC classification showed slightly higher performance with longer windows, reaching the highest *F*_1_-score at 15 seconds (0.742). However, bootstrap-based CI results showed substantial overlap across window lengths for both PB and AIC outcomes, indicating limited practical differences between configurations (Multimedia Appendix 7). Although longer windows yielded marginally higher AIC performance, differences across window lengths were small; therefore, the 10-second window was selected for subsequent analyses as a compromise between classification performance and temporal resolution.

#### Single-Sensor Model Performance Using activPAL Data

As shown in Table 3, for PB classification using activPAL data, the MH-CNN achieved the highest *F*_1_-score (0.716), outperforming RF models trained with CAPTURE-24 and the adapted HARTH features. For AIC classification, the RF models showed the best performance, with *F*_1_-scores of 0.736 (CAPTURE-24) and 0.738 (adapted HARTH), whereas the MH-CNN showed reduced performance (0.707).

**Table 3.** Physical behavior and activity intensity categories classification performance using activPAL data across machine learning and deep learning models.

ML^a^/DL^b^ approach	Precision	Recall	*F*_1_-score
PB^c^, mean (SD)			
RF^d^ and CAPTURE-24	0.646 (0.003)	0.775 (0.0004)	0.694 (0.002)
RF and HARTH (adapted)	0.639 (0.001)	0.777 (0.001)	0.690 (0.001)
MH-CNN^e^	0.698 (0.016)	0.744 (0.007)	0.716 (0.011)
AIC^f^, mean (SD)			
RF and CAPTURE-24	0.740 (0.001)	0.741 (0.001)	0.736 (0.001)
RF and HARTH (adapted)	0.741 (0.001)	0.746 (0.0002)	0.738 (0.001)
MH-CNN	0.726 (0.002)	0.723 (0.005)	0.707 (0.008)

a ML: machine learning

b DL: deep learning.

c PB: physical behavior.

d RF: random forest.

e MH-CNN: multihead convolutional neural network.

f AIC: activity intensity categories.

#### Single-Sensor Model Performance Using ActiGraph Data

As shown in Table 4, using ActiGraph data alone, PB classification performance was lower than that observed with activPAL. The MH-CNN achieved the highest *F*_1_-score (0.591), slightly outperforming RF models trained with CAPTURE-24 features and performing comparably to the RF model using adapted HARTH features.

**Table 4.** Physical behavior and activity intensity categories classification performance using ActiGraph data across machine learning and deep learning models.

ML^a^/DL^b^ approach	Precision	Recall	*F*_1_-score
PB^c^, mean (SD)			
RF^d^ and CAPTURE-24	0.535 (0.004)	0.688 (0.007)	0.553 (0.003)
RF and HARTH (adapted)	0.555 (0.002)	0.725 (0.002)	0.581 (0.002)
MH-CNN^e^	0.580 (0.006)	0.715 (0.008)	0.591 (0.004)
AIC^f^, mean (SD)			
RF and CAPTURE-24	0.718 (0.001)	0.734 (0.001)	0.716 (0.001)
RF and HARTH (adapted)	0.721 (0.0003)	0.737 (0.001)	0.721 (0.001)
MH-CNN	0.717 (0.002)	0.718 (0.006)	0.697 (0.006)

a ML: machine learning.

b DL: deep learning.

c PB: physical behavior.

d RF: random forest.

e MH-CNN: multihead convolutional neural network.

f AIC: activity intensity categories.

For AIC classification, RF models outperformed the MH-CNN. The adapted HARTH feature set achieved the highest *F*_1_-score (0.721), marginally above the CAPTURE-24 RF model. The MH-CNN yielded the lowest performance of the 3 approaches (*F*_1_-score=0.697). To obtain comparable results with ActiGraph data, the MH-CNN architecture required an increase in the number of CNN filters from 32 to 64, while all other hyperparameters were kept unchanged.

#### Dual-Sensor Model Performance

As shown in Table 5, when combining activPAL and ActiGraph data, performance improved for PB classification over single sensor configurations (Tables 3 and 4). The MH-CNN achieved the highest *F*_1_-score (0.750), outperforming the RF model trained with the dual-sensor HARTH feature set (*F*_1_-score=0.729). For AIC classification, the RF dual-sensor HARTH model achieved the highest and most stable performance (*F*_1_-score=0.741).

**Table 5.** Physical behavior and activity intensity categories classification performance using the dual-sensor (activPAL + ActiGraph) configuration.

ML^a^/DL^b^ approach	Precision	Recall	*F*_1_-score
PB^c^, mean (SD)			
RF^d^ and HARTH (dual-sensor)	0.671 (0.002)	0.825 (0.002)	0.729 (0.002)
MH-CNN^e^	0.720 (0.015)	0.797 (0.011)	0.750 (0.008)
AIC^f^, mean (SD)			
RF and HARTH (dual-sensor)	0.743 (0.002)	0.749 (0.001)	0.741 (0.002)
MH-CNN	0.734 (0.003)	0.734 (0.005)	0.722 (0.006)

a ML: machine learning.

b DL: deep learning.

c PB: physical behavior.

d RF: random forest.

e MH-CNN: multihead convolutional neural network.

f AIC: activity intensity categories.

Overall, dual-sensor configurations consistently outperformed single-sensor setups for PB classification, while variability across the 5 repeated runs remained low, indicating robust and stable model behavior. Although the RF model achieved the highest AIC classification performance, the overall multitask performance of the RF and MH-CNN models was comparable (mean *F*_1_-score between PB and AIC: 0.736 for MH-CNN and 0.735 for RF). To maintain a unified multitask framework across both outcomes, the MH-CNN was selected for subsequent analyses.

### Free-Living Daily PB and AIC Estimates

#### Model-Derived Daily PB Profiles Across Sensor Configurations

Following model development and results, the selected MH-CNN models were applied to the test subset data (n=87) to generate a (weekday-weekend) weighted average of daily PB estimates across the 9-day free-living monitoring period for the 3 sensor configurations (activPAL, ActiGraph, and dual-sensor).

**Figure 3.**
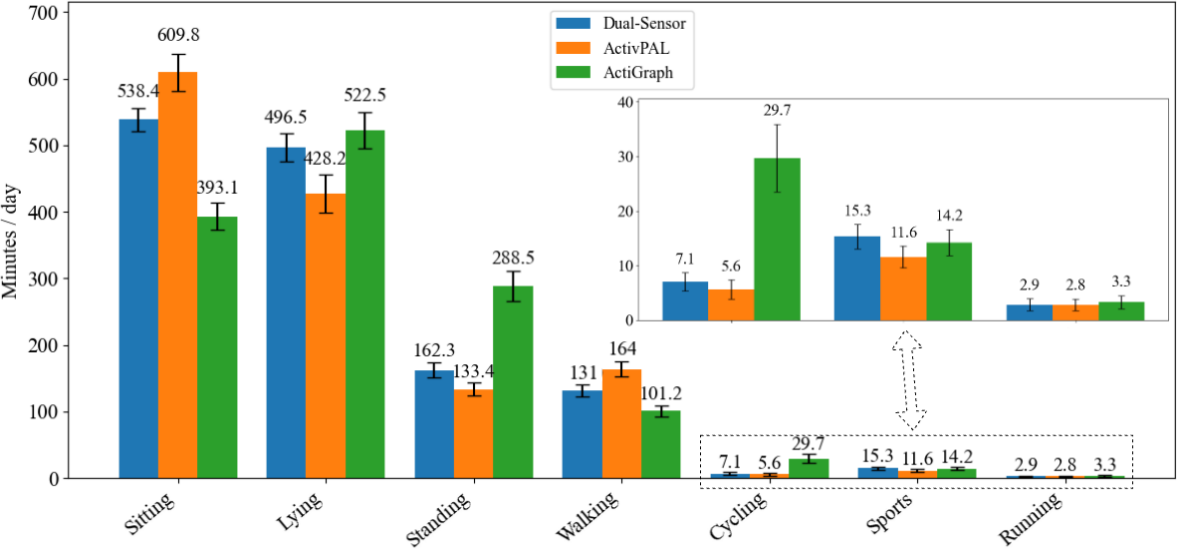
Weighted average daily distribution of time spent in physical behavior estimated using activPAL, ActiGraph, and dual-sensor configurations over 9 days. Error bars represent 95% CIs across participants.

As shown in Figure 3, sedentary behaviors, particularly sitting and lying, accounted for the highest proportion of daily time in all sensor modalities. The dual-sensor approach estimated 37% (538.4/1440 min) of the day as sitting and 34% (496.5/1440 min) as lying.

In comparison, our activPAL ML algorithm estimated 42% (609.8/1440 min) of the day as sitting and 30% (428.2/1440 min) as lying, while ActiGraph showed the opposite pattern, with 27% (393.1/1440 min) as sitting and 36% (522.5/1440 min) as lying. For standing time, the dual-sensor configuration estimated 11% (162.3/1440 min) of the day, yielding values intermediate between those obtained with the single-sensor devices. Specifically, activPAL estimated a lower standing time of 9% (133.4/1440 min), whereas ActiGraph produced higher estimates of 20% (288.5/1440 min).

A similar pattern was observed for walking. The dual-sensor model estimated 9% (131/1440 min) of the day, with values falling between those obtained from the single-sensor devices. ActivPAL yielded higher estimates (164/1440 min, 11%), while ActiGraph provided lower estimates (101.2/1440 min, 7%).

PB with low expected temporal prevalence under free-living conditions such as cycling (6–30/1440 min, <2%), sports (12–15/1440 min, ~1%), and running (~3/1440 min, <1%) accounted for only a small proportion of the daily activity profile. Among the single-sensor modalities, ActiGraph yielded higher estimates for these infrequent behaviors, most notably cycling (29.7/1440 min, 2%), whereas the dual-sensor approach produced lower estimates.

#### Model-Derived Daily AIC Profiles Across Sensor Configurations

Similarly, the selected MH-CNN models were applied to the test subset data to generate weighted average daily AIC estimates across the 9-day free-living monitoring period.

**Figure 4.**
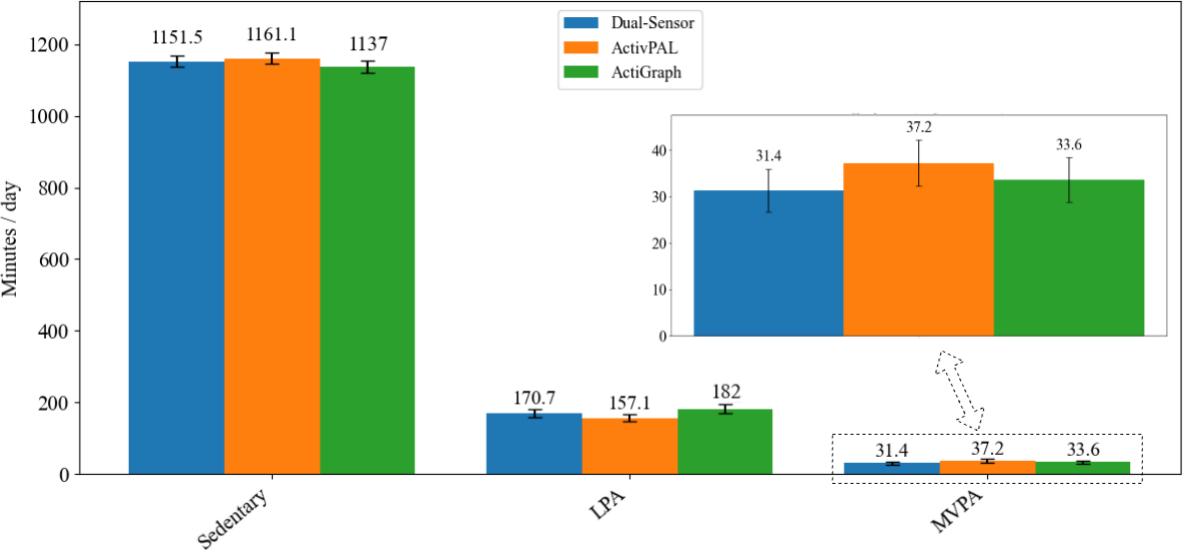
Weighted average daily distribution of time spent in activity intensity categories estimated using activPAL, ActiGraph, and dual-sensor configurations over 9 days. Error bars represent 95% CIs across participants. LPA: light physical activity; MVPA: moderate-to-vigorous physical activity.

As shown in Figure 4, estimated weighted average daily AIC exhibited the expected gradient across intensity categories, with sedentary time comprising most of the day across all sensor modalities.

The dual-sensor approach estimated 80% (1151.5/1440 min) of the day as sedentary behavior, with comparable values from activPAL (1161.1/1440 min, 81%) and ActiGraph (1137/1440 min, 79%). These higher totals reflect that waking sedentary time and sleep were merged into a single sedentary category.

For LPA, daily estimates ranged from 11%‐13% (157.1‐182.0/1440 min), with ActiGraph reporting the highest values (182/1440 min, 13%), activPAL the lowest (157.1/1440 min, 11%), and the dual-sensor configuration producing an intermediate estimate of 12% (170.7/1440 min).

MVPA contributed only a small portion of the daily time. The dual-sensor approach estimated 2% (31.4/1440 min), compared with 3% (37.2/1440 min) for activPAL, and 2% (33.6/1440 min) for ActiGraph.

#### Agreement Between Dual-Sensor Estimates and CREA for PBs

**Figure 5.**
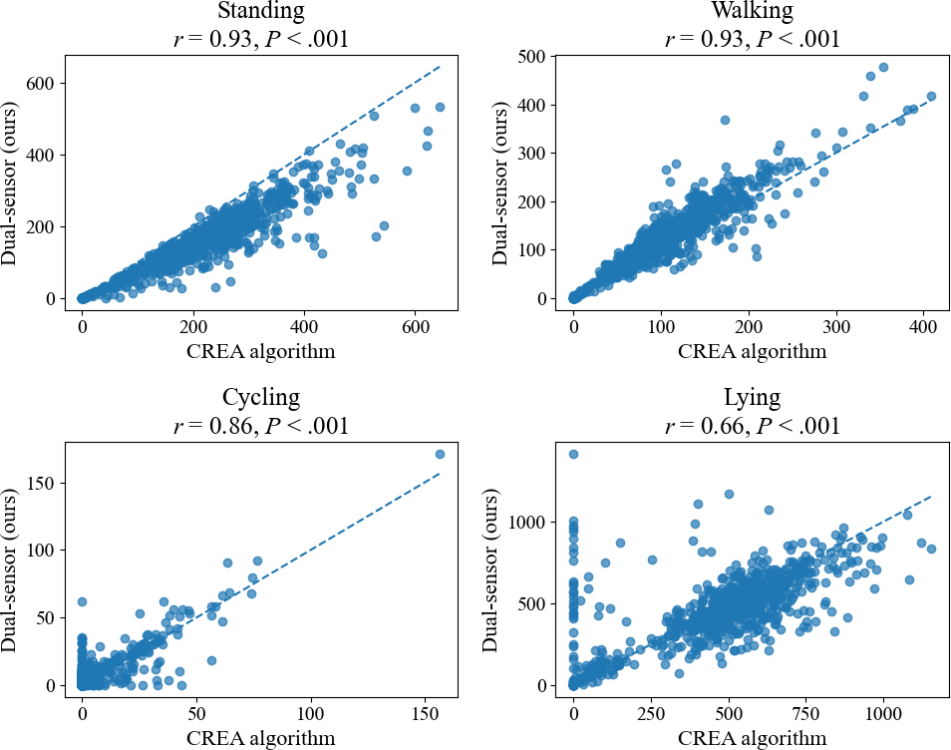
Correlations between classification of real-world everyday activities and dual-sensor estimates of daily time spent in physical behavior categories. Each point represents one participant-day, showing the daily duration (minutes/day) estimated by the classification of real-world everyday activities algorithm (x-axis) and the dual-sensor model (y-axis). Pearson coefficients (*r*) and *P* values are shown for each physical behavior. CREA: classification of real-world everyday activities.

**Figure 6.**
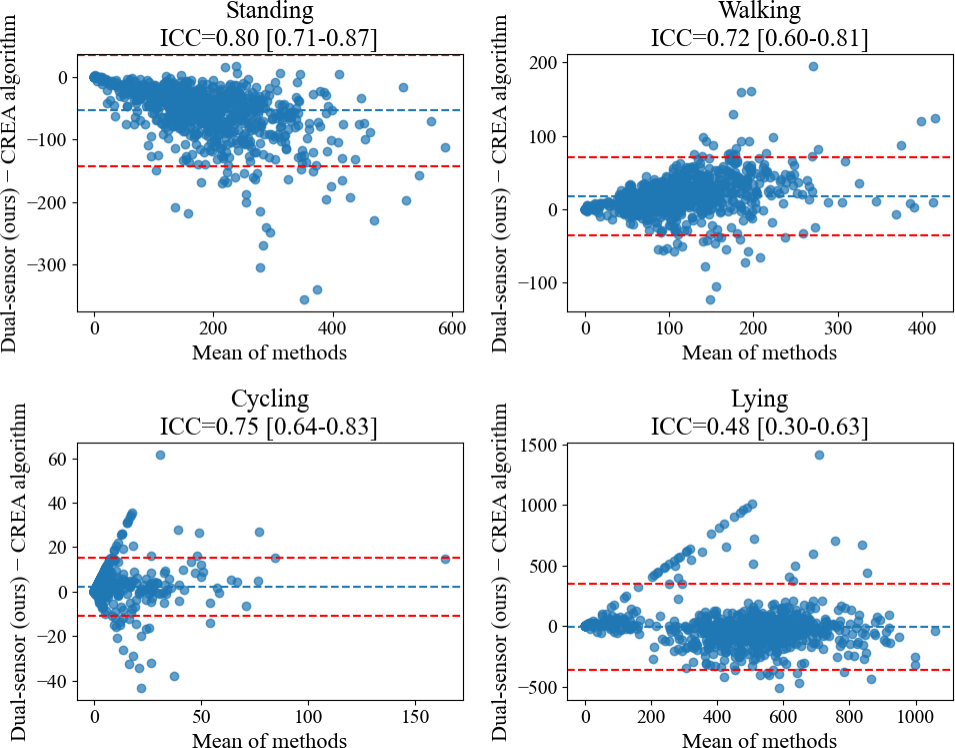
Bland-Altman plots showing agreement between classification of real-world everyday activities and dual-sensor estimates of daily time spent in physical behavior categories. Each point represents the difference between dual-sensor and classification of real-world everyday activities daily estimates plotted against their mean at daily level. Intraclass correlation coefficients with 95% CIs are reported for each physical behavior. CREA: classification of real-world everyday activities; ICC: intraclass correlation coefficients.

The dual-sensor configuration demonstrated varying levels of agreement with the CREA algorithm across the 4 PB categories, as illustrated in the correlation analyses (Figure 5) and Bland-Altman results (Figure 6). These analyses were based on participant-specific daily estimates, with each data point representing a direct day-by-day comparison between the 2 methods.

Standing and walking showed the strongest correspondence, with high correlations (*r*=0.93, *P*<.001 for both) and substantial ICC values (standing: ICC=0.80, 95% CI 0.71‐0.87; walking: ICC=0.72, 95% CI 0.60‐0.81). Bland-Altman plots indicated relatively narrow limits of agreement and minimal systematic bias.

Cycling also showed a strong correlation (*r*=0.86, *P*<.001) and good reliability (ICC=0.75, 95% CI 0.64‐0.83), although the Bland-Altman plot revealed greater dispersion at longer cycling durations.

Lying exhibited a similar pattern, with moderate correlation (*r*=0.66, *P*<.001) and fair reliability (ICC=0.48, 95% CI 0.30‐0.63) with increased dispersion at higher daily durations.

#### Agreement Between Dual-Sensor Estimates and CREA for AIC

**Figure 7.**
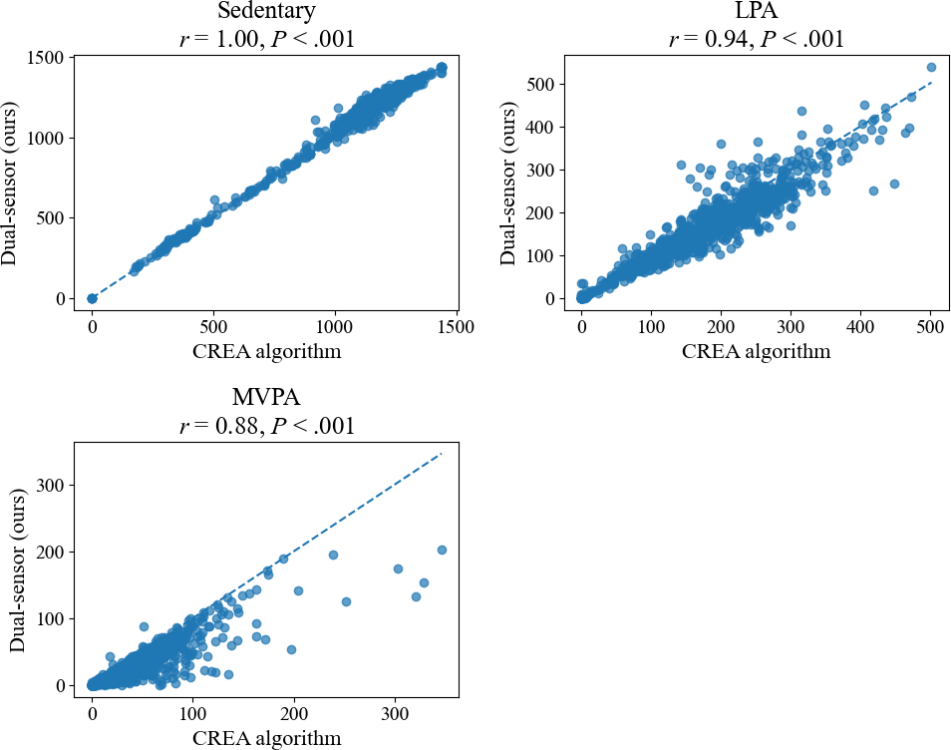
Correlations between classification of real-world everyday activities and dual-sensor estimates of daily time spent in AIC. Each point represents one participant-day, showing the daily duration (minutes/day) estimated by the classification of real-world everyday activities algorithm (x-axis) and the dual-sensor model (y-axis). Pearson coefficients (*r*) and *P* values are shown for each activity intensity category. CREA: classification of real-world everyday activities; LPA: light physical activity; MVPA: moderate-to-vigorous physical activity.

**Figure 8.**
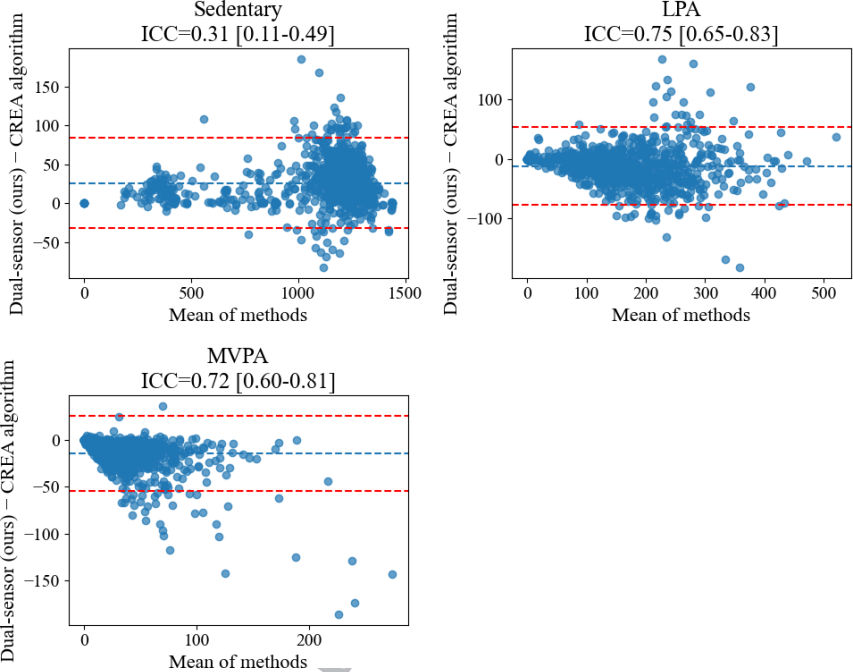
Bland-Altman plots showing agreement between classification of real-world everyday activities and dual-sensor estimates of daily time spent in activity intensity categories. Each point represents the difference between dual-sensor and classification of real-world everyday activities daily estimates plotted against their mean at the daily level. Intraclass correlation coefficients with 95% CIs are reported for each activity intensity category. CREA: classification of real-world everyday activities; ICC: intraclass correlation coefficient; LPA: light physical activity; MVPA: moderate-to-vigorous physical activity.

The agreement between the dual-sensor configuration and the CREA algorithm varied among AIC, as illustrated in the correlation (Figure 7) and Bland-Altman plots (Figure 8).

Sedentary showed a near-perfect correlation (*r*=1.00, *P*<.001; Figure 7). However, the Bland-Altman analysis (Figure 8) revealed a wide dispersion at higher sedentary levels, resulting in poor reliability (ICC=0.31, 95% CI 0.11‐0.49), with increased disagreement above approximately 1000 minutes/day.

For LPA, a strong correlation (*r*=0.94, *P*<.001) and good reliability (ICC=0.75, 95% CI 0.65‐0.83) were observed. MVPA also showed strong agreement (*r*=0.88, *P*<.001; ICC=0.72, 95% CI 0.60‐0.81), with Bland-Altman plots indicating an increasing negative bias at longer MVPA durations.

## Discussion

### Overview of Key Findings

This study provides a comprehensive development and evaluation of multitask ML models for simultaneous PB and AIC classification using multisensor accelerometry under free-living conditions. Leveraging data from 590 adults across 4 European countries monitored over a 9-day period, the findings demonstrate that integrating thigh- and waist-worn sensors within a multitask learning framework improves model performance and the stability of daily behavioral profiles compared with single-sensor approaches. Collectively, these results support the feasibility of scalable long-term wearable monitoring under real-world conditions, thereby providing ecologically valid evidence at a scale rarely reported in prior free-living wearable studies.

When applied to free-living data, the dual-sensor configuration produced PB distributions characterized by high sedentary time and comparatively limited engagement in ambulatory and vigorous behaviors. These estimates fall within ranges commonly reported in adult populations, where sedentary behaviors account for approximately 60%‐70% of daily time [31,43] and walking typically averages 1‐2 hours per day [44]. Similarly, low-prevalence behaviors such as running, sports, and cycling contributed minimally to daily activity, consistent with epidemiological observations that vigorous or sport-related activities occupy only a small fraction of daily time in adults [43,45].

For AIC, the dominance of sedentary time and the relative contributions of LPA and MVPA were consistent with population-based accelerometer studies reporting 8‐10 hours/day of sedentary behavior [46,47], 10%‐20% of time in LPA [48], and 20‐40 minutes/day of MVPA [39]. The dual-sensor configuration consistently produced intermediate estimates between single-sensor approaches, suggesting reduced sensor-specific bias and improved behavioral characterization across heterogeneous activity patterns. Similarly, the inclusion of participants with a broad range of BMI values, including individuals classified as obese, supported the representation of diverse PB and AIC patterns under free-living conditions.

Agreement analyses further showed that the dual-sensor approach aligned most closely with CREA for ambulatory behaviors (standing, walking, and cycling), while greater discrepancies were observed for prolonged sedentary behaviors, particularly lying. This pattern suggests that multimodal sensing enhances recognition of dynamic behaviors under free-living conditions but remains challenged by extended low-movement postures, a limitation also reported in prior work [49].

### Implications for Remote Monitoring

From a monitoring perspective, these findings have important implications for real-world implementation of wearable-based monitoring systems. First, they demonstrated that dual-sensor wearable monitoring could reduce modality-specific biases inherent to single-sensor approaches, particularly for posture-related behaviors. The dual-sensor configuration mitigated systematic over- and underestimation observed with activPAL-only and ActiGraph-only models, producing more balanced and interpretable daily PB profiles that are better suited for longitudinal monitoring and population-level analyses. The scalability of the dual-sensor approach warrants consideration for large-scale deployment. Although the use of 2 devices may increase participant burden, it proved feasible and was well accepted in our sample [50]. Furthermore, jointly labeled thigh- and hip-worn data enable the development of more accurate and generalizable models that can subsequently be applied in single-sensor configurations, supporting scalable mHealth apps [27].

Second, the integration of EMA-informed labeling provided a scalable and pragmatic solution to overcome the scarcity of reliable free-living training data. Event-based EMA enabled the collection of relevant behavior labels at the moment of occurrence, thereby improving ecological validity, supporting the development of models that generalize beyond controlled laboratory environments [23,24,26]. This approach is particularly relevant for mHealth studies requiring extended monitoring periods, where intensive manual annotation is not feasible.

Third, the relative stability of AIC estimates across sensor configurations suggests that multitask learning may be especially valuable for digital health apps focused on energy balance, PA guidelines, and surveillance of activity intensity distributions. Even when discrepancies were observed in PB classification, aggregated AIC estimates remained consistent with epidemiological norms reported in large cohort studies [39,46-48], supporting their use in public health monitoring and evaluation of intervention outcomes.

### Comparison With Prior Work

Previous studies on wearable-based activity recognition have frequently relied on laboratory-based protocols, short-term recordings, or single-sensor configurations, which may limit generalizability to free-living settings [22,25,26]. Although datasets such as CAPTURE-24 have advanced free-living activity recognition using video-based behavioral labels [16], reliance on wearable cameras and manual annotation may reduce scalability and raise privacy concerns for longer-term monitoring. In contrast, this study uses 9-day free-living data and EMA-informed labeling from a multicenter cohort, providing a rigorous assessment of model performance under realistic conditions.

Previous research has demonstrated the feasibility of EMA for behavioral annotation and the potential of multitask learning for activity recognition [17,18]. Extending this work, this study combined multitask learning with multisensor accelerometry in a large multicenter dataset, offering empirical evidence of their combined value for mHealth monitoring in real-world settings.

The findings are consistent with previous studies reporting that feature-engineered ML models can remain competitive with DL approaches for free-living activity recognition [21]. In this study, the MH-CNN achieved the highest PB classification performance, whereas the RF model achieved the highest AIC classification performance, suggesting that engineered temporal and statistical features remain informative for AIC classification under free-living conditions [25]. Although more complex sequential architectures may further improve temporal modeling, the selected MH-CNN offered a balanced compromise between predictive performance, complexity, and scalability for long-term monitoring.

### Limitations

Several limitations should be acknowledged. AIC estimates were derived from the activPAL CREA algorithm and operationalized using MET-derived categories rather than direct kcal-based energy expenditure, thereby limiting the assessment of continuous energy expenditure. In addition, the CREA algorithm contributed to the label-refinement procedure and was subsequently used as a comparator for daily estimates; therefore, comparisons with CREA-derived estimates represent assessments of agreement rather than independent validation. Although gold-standard methods such as doubly labeled water or other forms of indirect calorimetry would provide stronger absolute validation of the AIC estimates [3], these approaches were beyond the scope of this study and warrant further investigation within multitask wearable monitoring frameworks.

The behavioral taxonomy was limited to 7 PB categories and 3 activity intensity levels, which, although appropriate for large-scale monitoring, do not capture the full diversity of real-world activities. In addition, the EMA-labeled dataset was imbalanced, with low-frequency behaviors such as running and sports remaining underrepresented despite mitigation strategies including selective undersampling and class-weighted losses. Future research may benefit from data augmentation, self-supervised learning, or transfer learning approaches trained on large unlabeled datasets to improve recognition of infrequent behaviors [15].

Furthermore, although the MH-CNN improved PB classification performance, the comparatively smaller gains observed for AIC classification may reflect class imbalance and limitations in CNN-based temporal modeling under free-living conditions. Future work should evaluate sequential architectures such as LSTM- or Transformer-based models using larger (real-world) datasets. Finally, although the dual-sensor configuration improved performance, practical deployment in some mHealth contexts may require adaptation to single-sensor or consumer-grade devices, which warrants further investigation.

### Conclusions

This study demonstrates that multitask ML models combining thigh- and waist-worn accelerometry can provide consistent estimates of PB and AIC under free-living conditions. The dual-sensor approach produced stable, epidemiologically coherent daily profiles and reduced modality-specific biases observed in single-sensor methods. By integrating multimodal wearable sensing with EMA-informed labeling at scale, this work advances methodological foundations for real-world mHealth monitoring and supports more consistent and comparable assessment of PB in population and public health research.

## Supplementary material

10.2196/94302 Multimedia Appendix 1 Overview of the body-worn wearables, data sources, and processing workflow.

10.2196/94302 Multimedia Appendix 2 Characteristics of the sparsely EMA-labeled data before and after applying this data selection.

10.2196/94302 Multimedia Appendix 3 Description of the set of features extracted.

10.2196/94302 Multimedia Appendix 4 Details of the hyperparameter tuning.

10.2196/94302 Multimedia Appendix 5 Parameters of the multihead convolutional neural network.

10.2196/94302 Multimedia Appendix 6 Characteristics of the data subsets used for training and evaluation.

10.2196/94302 Multimedia Appendix 7 Bootstrap-based 95% CIs for macro *F*_1_-scores across evaluated window lengths.

## Abbreviations

AIC: activity intensity categories
CREA: classification of real-world everyday activities (activPAL algorithm)
DL: deep learning
EMA: ecological momentary assessment
ICC: intraclass correlation coefficient
LPA: light physical activity
MET: metabolic equivalent of task
MH-CNN: multihead convolutional neural network
mHealth: mobile health
ML: machine learning
MVPA: moderate-to-vigorous physical activity
PA: physical activity
PB: physical behavior
RF: random forest
SMOTE: synthetic minority oversampling technique
WEALTH: wearable sensor assessment of physical and eating behaviors
