# Supplementary material for: Robust Assessment of Free-Living Physical Behaviors and Activity Intensity Using Dual-Wearable Multitask Learning: Development and Evaluation Study From the Multicenter WEALTH Project
Source: JMIR Mhealth Uhealth. 2026 Aug 14;14:e94302. doi: 10.2196/94302 (PMC13481161; doi:10.2196/94302)
Supplement: Supplementary file 1 [file mhealth-v14-e94302-s001.docx]

# Multimedia Appendix

Figure S1: Overview of the body-worn wearables, EMA-based labelling strategy, and data processing workflow used for physical behaviour and activity intensity assesment.
