# Supplementary material for: Robust Assessment of Free-Living Physical Behaviors and Activity Intensity Using Dual-Wearable Multitask Learning: Development and Evaluation Study From the Multicenter WEALTH Project
Source: JMIR Mhealth Uhealth. 2026 Aug 14;14:e94302. doi: 10.2196/94302 (PMC13481161; doi:10.2196/94302)
Supplement: Supplementary file 2 [file mhealth-v14-e94302-s002.docx]

# Multimedia Appendix

## Characteristics of the sparsely EMA-labelled data before and after applying a threshold-based data selection

Table S1: Characteristics of EMA sparsely labelled data before and after applying a 75% segment selection threshold.

| **Activity** | **Segment Accuracy (%), mean (SD)** | **Data in hours** | **Segment Accuracy (%), mean (SD)** | **Data in hours** |
| --- | --- | --- | --- | --- |
|  | **No selection** | **No selection** | **75% threshold** | **75% threshold** |
| Cycling | 51.5 (37.0) | 41.5 | 91.1 (7.9) | 13 |
| Lying | 59.0 (48.9) | 443.9 | 99.7 (2.0) | 268.7 |
| Running | 94.4 (14.6) | 13.6 | 97.2 (5.6) | 12.9 |
| Sitting | 87.3 (30.7) | 1837.6 | 99.0 (3.3) | 1584.7 |
| Sports | 69.2 (30.2) | 22.1 | 92.0 (8.6) | 9.9 |
| Standing | 44.8 ( 45.8) | 98.1 | 97.7 ( 4.2) | 40.4 |
| Walking | 74.9 ( 25.9) | 339.2 | 90.6 ( 8.3) | 178.5 |

Before quality control, a total of 2796 hours (h) of EMA-labelled data were generated from 590 participants. Sitting was the most prevalent behaviour (1837.6 h; 5507 segments), followed by lying (443.9 h; 1369 segments) and walking (339.2 h; 3538 segments), while standing (98.1 h; 294 segments), running (94.4 h; 165 segments), cycling (41.5 h; 445 segments), and sports (22.1 h; 215 segments) were less frequent.

Prior to thresholding, mean segment-level accuracy varied across behaviours: standing 44.8% (SD 45.8%), cycling 51.5% (SD 37.0%), lying 59.0% (SD 48.9%), sports 69.2% (SD 30.2%), walking 74.9% (SD 25.9%), sitting 87.3% (SD 30.7%), and running 94.4% (SD 14.6%).

After implementing the 75% agreement threshold, a total of 2108 h of labelled data from 583 participants were retained for machine learning training and validation. This procedure substantially improved the average accuracy across all behaviours, with overall segment-level accuracies exceeding 90% and approaching near-perfect levels for sitting 99% (SD 3.3) and lying 99.7% (SD 2%). This improvement was achieved at the expense of reduced data amount. The final distribution of the retained data across physical behaviours (PB) was as follows: sitting 75.2% (1584.7/2108 h), lying 12.7% (268.0/2108 h), walking 8.5% (178.5/2108 h), standing 1.9% (40.4/2108 h), cycling 0.6% (13.0/2108 h), running 0.6% (12.9/2108 h), and sports 0.5% (9.9/2108 h).
