# Supplementary material for: Robust Assessment of Free-Living Physical Behaviors and Activity Intensity Using Dual-Wearable Multitask Learning: Development and Evaluation Study From the Multicenter WEALTH Project
Source: JMIR Mhealth Uhealth. 2026 Aug 14;14:e94302. doi: 10.2196/94302 (PMC13481161; doi:10.2196/94302)
Supplement: Supplementary file 3 [file mhealth-v14-e94302-s003.docx]

# Multimedia Appendix

### CAPTURE-24 feature set

A set of 40 features proposed in the CAPTURE-24 [1] study were computed from the tri-axial accelerometer signals. Table S2 summarizes the features extracted.

Table S2. CAPTURE-24 feature set.

| **Group of features** | **Description** | **Features** |
| --- | --- | --- |
| Quantiles | Minimum, maximum, median, 25th and 75th percentil of acceleration for each of the three axes and for the resultant magnitude vector in the time domain. | 20 |
| Correlations | Correlation between axes and 1-second lag autocorrelation of the magnitude vector. | 4 |
| Spectral features | Spectral entropy; first and second dominant frequencies and their corresponding powers. | 5 |
| Peak features | Number of peaks and median prominence of the peaks. | 2 |
| Angular features | Estimated dynamic roll, pitch and yaw (mean and standard deviation), and gravity-based roll, pitch, and yaw (reported as mean) | 9 |

### HARTH feature set adapted for single tri-axial sensors

An adapted set of 71 features, originally proposed in the HARTH [2] study, were computed from the tri-axial accelerometer signals. The gravity component was estimated by computing the sum-vector of the tri-axial signal and subsequently applying a fourth-order low-pass Butterworth filter with a cut-off frequency of 1 Hz. Table S3 summarizes the features extracted.

Table S3. HARTH features adapted for single tri-axial sensors. FFT: Fast Fourier Transform.

| **Group of features** | **Description** | **Features** |
| --- | --- | --- |
| Time-domain features for each axis (3) and the (1) gravity component | Standard time-domain features extracted from acceleration signals. This group includes 8 features per axis (e.g., mean, median, standard deviation, coefficient of variation, percentile 25, percentile 75, min, max). | 32 |
| Movement components for each axis (3) and the (1) gravity component | Common movement components extracted from the time and frequency domain: (3) Time (skew, kurtosis, signal energy); (9) Frequency (FFT mean, FFT standard deviation, dominant frequency; dominant frequency-magnitude, spectral centroid, total power). | 36 |
| 3-axis correlation features | Pairwise correlation coefficients computed across the three movement axes. | 3 |

### HARTH feature set for dual-sensors

A set of 161 features proposed in the HARTH [2] study were computed from the tri-axial accelerometer signals. The gravity components for each sensor were estimated by computing the sum-vector of each tri-axial signal and subsequently applying a fourth-order low-pass Butterworth filter with a cut-off frequency of 1 Hz. Table S4 summarizes the features extracted.

Table S4. HARTH features for dual sensors. FFT: Fast Fourier Transform.

| **Group of features** | **Description** | **Features** |
| --- | --- | --- |
| Time-domain features for each axis (6) and the (2) gravity components of each sensor | Standard time-domain features extracted from acceleration signals. This group includes 8 features per axis (e.g., mean, median, standard deviation, coefficient of variation, percentile 25, percentile 75, min, max). | 64 |
| Movement components for each axis (6) and the (2) gravity component | Common movement components extracted from the time and frequency domain: (3) Time (skew, kurtosis, signal energy); (9) Frequency (FFT mean, FFT standar deviation, dominant frequency; dominant frequency-magnitude, spectral centroid, total power). | 72 |
| 6-axis correlation features | Pairwise correlation coefficients computed across the six movement axes, accounting for all 15 possible axis combinations. | 15 |
| Correlation magnitude | Correlation between the magnitude vectors of the two sensors. | 1 |
| Mean acceleration (gravity-based) | Mean acceleration features computed by averaging the gravity components between the two sensors. | 9 |

# References

1. Chan, S. *et al.* Capture-24: A large dataset of wrist-worn activity tracker data collected in the wild for human activity recognition. *Sci. Data* **11**, 1135 (2024).
2. Logacjov, Aleksej, et al. "HARTH: a human activity recognition dataset for machine learning." *Sensors* **21**.23 (2021): 7853.
