# Supplementary material for: Robust Assessment of Free-Living Physical Behaviors and Activity Intensity Using Dual-Wearable Multitask Learning: Development and Evaluation Study From the Multicenter WEALTH Project
Source: JMIR Mhealth Uhealth. 2026 Aug 14;14:e94302. doi: 10.2196/94302 (PMC13481161; doi:10.2196/94302)
Supplement: Supplementary file 4 [file mhealth-v14-e94302-s004.docx]

# Multimedia Appendix

## Hyperparameter tuning details

Table S5. Range of values and steps used for the optimization of the machine and deep learning approaches.

| **Model** | **Parameter** | **Range** | **Step** |
| --- | --- | --- | --- |
|  | Number of trees | [50, 100, 150] |  |
| Random forest | Max depth | 5 to 10 | 1 |
|  | Max features | 10 to 20 | 1 |
|  | Learning rate | 1*·*10*^−^*^5^ to 1*·*10*^−^*^1^ | Continuous value |
|  | Batch size | [64, 128, 256, 512, 1024, 2048] |  |
| MH-CNN | Number of CNN filters | [16, 32, 64, 96, 128] |  |
|  | Dropout CNN | 0.1 to 0.5 | 0.1 |
|  | Dropout Dense | 0.1 to 0.5 | 0.1 |
