# Supplementary material for: Robust Assessment of Free-Living Physical Behaviors and Activity Intensity Using Dual-Wearable Multitask Learning: Development and Evaluation Study From the Multicenter WEALTH Project
Source: JMIR Mhealth Uhealth. 2026 Aug 14;14:e94302. doi: 10.2196/94302 (PMC13481161; doi:10.2196/94302)
Supplement: Supplementary file 5 [file mhealth-v14-e94302-s005.docx]

# Multimedia Appendix

## Multi-Head Convolutional Neural Network and training settings

Table S6. Summary of the parameters of the multi-head convolutional neural network (MH-CNN). f=filters; k=kernel; dl=dilatation rate; dp=dropout rate; p=pool size; u=units; GAP=global average pooling

| **Layer** | **Parameters** | **Output Shape** | **Parameters** |
| --- | --- | --- | --- |
| Input | - | (200, 6) | 0 |
| Spatial dropout | dp=0.1 | (200,6) | 0 |
| CNN1 (medium size branch) | f=32, k=6, dl=1 | (195, 32) | 1184 |
| Dropout (medium size branch) | dp=0.4 | (195, 32) | 0 |
| Max pooling (medium size branch) | p=2 | (97,32) | 0 |
| CNN2 (medium size branch) | f=32, k=6, dl=2 | (87, 32) | 6176 |
| Dropout (medium size branch) | dp=0.4 | (87, 32) | 0 |
| Max pooling (medium size branch) | p=2 | (43,32) | 0 |
| GAP (medium size branch) | - | (32) | 0 |
| CNN1 (long size branch) | f=32, k=9, dl=1 | (192, 32) | 1760 |
| Dropout (long size branch) | dp=0.4 | (192, 32) | 0 |
| Max pooling (long size branch) | p=2 | (96,32) | 0 |
| CNN2 (long size branch) | f=32, k=9, dl=2 | (80, 32) | 9248 |
| Dropout (long size branch) | dp=0.4 | (80, 32) | 0 |
| Max pooling (long size branch) | p=2 | (40,32) | 0 |
| GAP (long size branch) | - | (32) | 0 |
| Concatenation | - | (64) | 0 |
| Dense (task 1) | u=32 | (32) | 2080 |
| Dropout (task 1) | dp=0.4 | (32) | 0 |
| Softmax(task 1) | u=7 | (7) | 231 |
| Dense (task 2) | u=32 | (32) | 2080 |
| Dropout (task 2) | dp=0.4 | (32) | 0 |
| Softmax (task 2) | u=3 | (3) | 99 |
| Total params |  |  | 22858 |

The multi-head convolutional neural network (MH-CNN) architecture was trained with a learning rate of 3×10⁻⁴, batch size of 512, and 120 epochs. Early stopping was applied by monitoring the validation loss, using a minimum improvement threshold of 1×10⁻⁶ and patience of five epochs, to mitigate the risk of overfitting. Optimisation used the Adaptive Moment Estimation (ADAM) optimizer with eight warm-up steps to stabilise early gradient updates and prevent divergence during the initial training phase. The model followed a multitask learning framework with task-specific weights prioritising physical behaviour (PB) classification over actitvity intensity categories (AIC) classification (ratio 10:1). This ratio was determined empirically by testing different values to achieve optimal performance in both tasks. PB classification was assigned a higher weight because it is a more challenging task, with greater variability and a larger number of activity classes. Class-weighted losses were also applied within each task to mitigate the remaining imbalance in both PB and AIC tasks. Weights for each class were computed using the data distribution of the training data.
