# Supplementary material for: Robust Assessment of Free-Living Physical Behaviors and Activity Intensity Using Dual-Wearable Multitask Learning: Development and Evaluation Study From the Multicenter WEALTH Project
Source: JMIR Mhealth Uhealth. 2026 Aug 14;14:e94302. doi: 10.2196/94302 (PMC13481161; doi:10.2196/94302)
Supplement: Supplementary file 6 [file mhealth-v14-e94302-s006.docx]

# Multimedia Appendix

## Characteristics of the data subsets used for training and evaluation

Table S7: Data subsets used to train and evaluate the machine learning algorithms (N=583).

| **Characteristics** | | **Training** | **Validation** | **Test** |
| --- | --- | --- | --- | --- |
| Participants (male:female), n | | 409 (177:232) | 87 (39:48) | 87 (39:48) |
| Age, mean (SD) | | 38.1 (14.4) | 37.9 (14.5) | 37.9 (14.0) |
| **Participants per country, n** | |  |  |  |
|  | Czechia | 88 | 25 | 23 |
|  | France | 105 | 16 | 18 |
|  | Germany | 106 | 23 | 23 |
|  | Ireland | 110 | 23 | 23 |
