# Supplementary material for: Robust Assessment of Free-Living Physical Behaviors and Activity Intensity Using Dual-Wearable Multitask Learning: Development and Evaluation Study From the Multicenter WEALTH Project
Source: JMIR Mhealth Uhealth. 2026 Aug 14;14:e94302. doi: 10.2196/94302 (PMC13481161; doi:10.2196/94302)
Supplement: Supplementary file 7 [file mhealth-v14-e94302-s007.docx]

# Multimedia Appendix

**Bootstrap-based 95% confidence intervals for macro *F*_1_-scores across evaluated window lengths.**

Table S8: Bootstrap-based 95% confidence intervals for macro *F*_1_-scores across evaluated window lengths for physical behaviour (PB) and activity intensity categories (AIC) classification. Values represent the macro *F*_1_-score, reported as the mean bootstrap estimate with corresponding 95% confidence intervals (CI) obtained from 100 bootstrap resamples.

| **Window** | **PB *F*_1_-score (95% CI)** | **AIC *F*_1_-score (95% CI)** |
| --- | --- | --- |
| 5 s | 0.734 (95% CI 0.731–0.739) | 0.728 (95% CI 0.726–0.731) |
| 10 s | 0.731 (95% CI 0.726–0.738) | 0.740 (95% CI 0.737–0.744) |
| 15 s | 0.732 (95% CI 0.726–0.738) | 0.742 (95% CI 0.737–0.747) |
